# Legacy Building in Pediatric End-of-Life Care through Innovative Use of a Digital Stethoscope

**DOI:** 10.1089/pmr.2020.0028

**Published:** 2020-08-06

**Authors:** Elyse Andrews, Amelia Hayes, Laura Cerulli, Elissa G. Miller, Nicholas Slamon

**Affiliations:** ^1^ Thomas Jefferson University Pediatrics Residency Program, Nemours/Alfred I. duPont Hospital for Children, Wilmington, Delaware, USA.; ^2^ Division of Palliative Medicine, Department of Pediatrics, Nemours/Alfred I. duPont Hospital for Children, Wilmington, Delaware, USA.; ^3^ Department of Child Life, Nemours/Alfred I. duPont Hospital for Children, Wilmington, Delaware, USA.; ^4^ Sidney Kimmel Medical College, Thomas Jefferson University, Philadelphia, Pennsylvania, USA.; ^5^ Division of Critical Care Medicine, Department of Pediatrics, Nemours/Alfred I. duPont Hospital for Children, Wilmington, Delaware, USA.

**Keywords:** bereavement, child life specialist, end-of-life, legacy making, mementos, memory making, music therapy, palliative care, parental/caregiver grief, pediatric

## Abstract

***Background:*** Legacy making has been the focus of recent literature; however, few studies examine how legacy making affects bereaved parents.

***Objective:*** To better understand legacy making's effect on bereaved parents, this study examined (1) the presentation of legacy making to parents, (2) parent satisfaction, and (3) parent utilization of the project.

***Design:*** Eko CORE (Eko Devices, Inc., Berkeley, CA), a digital stethoscope that generates a phonocardiogram, a graphical representation of S1 and S2 heart sounds, was used to record children's heartbeats as they approached end of life. The heartbeat was then overlaid to a song or voice recording or kept as a stand-alone file. An artistic embellishment of the phonocardiogram was also created. Parents were surveyed about their experience with the Music Therapy Heart Sounds (MTHS) program. Twelve parents completed the survey.

***Setting/subjects:*** Tertiary care children's hospital. The subjects were bereaved parents.

***Measurements:*** Five-question survey. Institutional Review Board review exempt.

***Results:*** All respondents would recommend the MTHS program to other families experiencing end-of-life decision making. Forty-two percent (*N* = 5) heard about the program from pediatric palliative physicians, and 50% (*N* = 6) heard about it from therapists such as music or child life. The respondents varied in how often they utilized their child's heartbeat recordings: 25% (*N* = 3) viewed or listened monthly, 33% (*N* = 4) not at all, 17% (*N* = 2) almost weekly, 17% less than monthly, and 8% (*N* = 1) daily.

***Conclusion:*** The MTHS program is an easy-to-implement and cost-effective way to perform legacy making that bereaved parents recommend for other families.

## Introduction

The death of a loved one forever changes one's life, and the emotional toll that the loss can take on families is immense. Multiple sources have asserted that the grief related to the loss of a child is more severe than that of a spouse or other family member.^1–7^ Several studies have reported that between 12% to 63% of bereaved parents experience prolonged grief,^8–10^ defined as intense sorrow and yearning lasting longer than six months or a year that leads to functional impairment in one's life.^11,12^

Given the severity of bereaved parent grief, the need for a multidisciplinary approach to aid parents and families in the grieving process is of paramount importance. In end-of-life care, the multidisciplinary team often includes the primary and subspecialty physicians, palliative care physicians, nursing staff, pastoral care, and the child life team. Recent research has explored the efficacy of adding music therapy to this multidisciplinary team. Music therapy has been shown to decrease negative psychological outcomes such as depression and anxiety that palliative care seeks to address.^13–15^

Another focus of recent literature is legacy making. Legacy making is the act of creating or doing something with the intention of remembrance. Pediatric health care facilities typically offer a wide variety of legacy-making activities such as patient hand molds, saving locks of hair, memory books, photography, writing (e.g., letters, poetry, and songs), and videos.^16–18^ These mementos have been well received by both patients and their families.^19^

Legacy making may be beneficial to both patients and their loved ones in a variety of ways from facilitating conversations about remembrance to helping family members come to terms with a loved one's mortality.^4,19,20^ A study by Chochinov et al. explored the effect of a writing intervention for adult patients near the end of their lives in which patients were asked about what mattered most to them, and the written transcript was given to an important relative. The act of writing about their circumstances reduced the patients' depressive symptoms.^19^ Schaefer et al. examined the effect of a legacy artwork project on caregivers' psychological well-being. Compared with the control, parents that participated in the artwork project reported fewer symptoms of prolonged grief and a greater sense of support from the hospital.^3,4^

Recent pediatric research has focused on the effect of legacy making on children and its effect on the family as a whole.^20–22^ In addition, the rise of technology has allowed for the creation of new and innovative ways to create legacy items. This study examined how legacy making was presented to parents of dying children and their perceptions of the activity.

## Methods

### Device/procedure

The Eko CORE (Eko Devices, Inc., Berkeley, CA, USA) is a digital stethoscope that has the ability to augment auscultated heart sounds up to 40 × , save recorded heart sounds in .wav files, live stream heart sounds to a remote examiner for telemedicine use, and create a phonocardiogram, a graphic representation of S1 and S2 sounds as well as various murmurs. For this study, the Eko CORE was used in a novel way to help create a digital keepsake for surviving family members by recording the heart sounds of dying children.

**FIG. 1.**
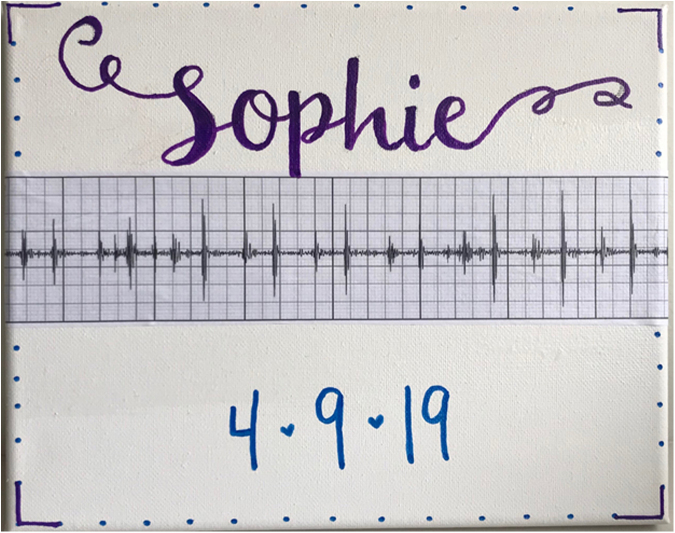
Sample artwork surrounding sample cardiac phonocardiogram.

**FIG. 2.**
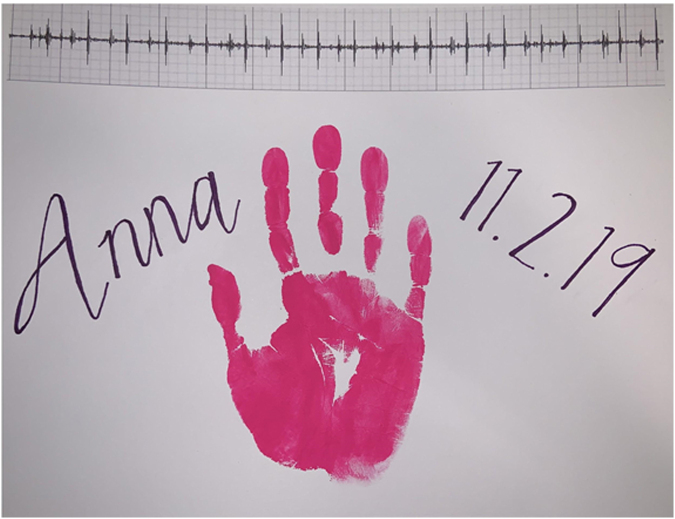
Sample artistic embellishment of a phonocardiogram.

For patients approaching the end of life, Music Therapy was consulted to record the child's heartbeat and edit ambient noise to isolate the heartbeat. The heartbeat was overlaid to a song or voice recording of the family or child or kept as a stand-alone file. Child life specialists and art therapists also created an artistic embellishment of the phonocardiogram (Figs. 1 and 2). The process was dubbed “the Music Therapy Heart Sounds (MTHS) program.” A survey to query parents about their experience was created with special interest in how they used the heartbeat recordings and how they were meaningful to them and their thoughts on making this a standard offering to families facing similar end-of-life circumstances. The survey was developed in conjunction with experts in the field, including palliative care and pediatric intensive care physicians, a music therapist, and a child life specialist. Our study was reviewed by our hospital's Institutional Review Board and was deemed exempt from further review.

### Participants

Eligible participants included parents of children who participated in the MTHS program between March 2018 and April 2019. The children were treated at our hospital for a variety of medical conditions. Exclusion criteria consisted of non-English-speaking families, families whose child was still living, families whose child passed away less than four months before the study period, those who had yet to receive a copy of their child's heart sounds, and anyone under investigation for nonaccidental trauma at the time of the child's death.

Sixteen families met the inclusion criteria. The parents and their contact information were identified through the electronic medical record. An e-mail that included an introduction letter to the study along with the five survey questions (Table 1) was sent to the eligible families. An online surveying platform was used to administer the survey. The survey included four multiple-choice and one open-text format questions. The multiple-choice questions had an “other” option in the event respondents could not find a choice that fit their desired response. Follow-up calls were made two weeks after the initial phone call. Three families were not able to be reached. Quantitative counting was used for the analysis of the four multiple-choice questions.

**Table 1.** Survey Questions

How did you hear about the MTHS program?
Do you think the MTHS program should be a standard offering to all patients who are experiencing end-of-life decision making?
Do you listen to or look at your child's heartbeat recording? Please understand that there is no correct answer for any individual family; as part of this survey we are simply trying to gather data.
In what other ways have you used your child's heartbeat recording?
In what ways has the MTHS recording program been most meaningful to you?

MTHS, Music Therapy Heart Sounds.

## Results

Eighty-five percent (*N* = 11) of the families who were able to be reached through telephone or e-mail completed the survey. Table 2 describes the demographics of the 12 parents who completed the survey. Most respondents were the mothers of the children (83%, *N* = 10). One family had both the mother and father of the child respond. Caucasian was the most common ethnicity (50%, *N* = 6), and a Christian denomination was the most common religion identified (58.3%, *N* = 7). The children were an average of seven years old at the time of death (range 8 days to 16 years). The families were contacted an average of 228 days after their child's death (range 105–411 days).

**Table 2.** Demographics of Respondents and Their Deceased Children

Relationship to child	
Mother	83% (*N* = 10)
Father	17% (*N* = 2)
Ethnicity	
Caucasian	50% (*N* = 6)
African American	17% (*N* = 2)
Hispanic	33% (*N* = 4)
Marital status	
Married	50% (*N* = 6)
Not married	50% (*N* = 6)
Religion	
Christian	58.3% (*N* = 7)
No religion listed	41.7% (*N* = 5)
Average amount of time since child's death (days)	228 (range 105–411)
Average age of child at time of death	7 Years (range 8 days–16 years)
Proposed cause of death	
Hypoxic ischemic encephalopathy	9% (*N* = 1)
Sepsis	18% (*N* = 2)
Status asthmaticus	9% (*N* = 1)
Status epilepticus	9% (*N* = 1)
Leptomeningeal glioneuronal tumor	9% (*N* = 1)
Acute lymphoblastic leukemia	9% (*N* = 1)
Graft versus host disease (IPEX syndrome underlying disease)	9% (*N* = 1)
Hypoplastic left heart syndrome	18% (*N* = 2)
Epidermolysis bullosa	9% (*N* = 1)

**FIG. 3.**
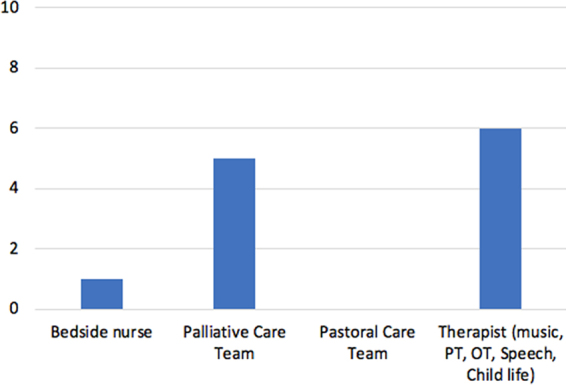
Graph showing how families heard about the heartbeat recording program.

Forty-two percent (*N* = 5) of the families learned about the MTHS program through the pediatric palliative care physicians. Another 50% (*N* = 6) of the families learned about the program from music, physical, occupational, speech, or child life specialists. One family discovered the MTHS program after a conversation with their bedside nurse (Fig. 3).

**FIG. 4.**
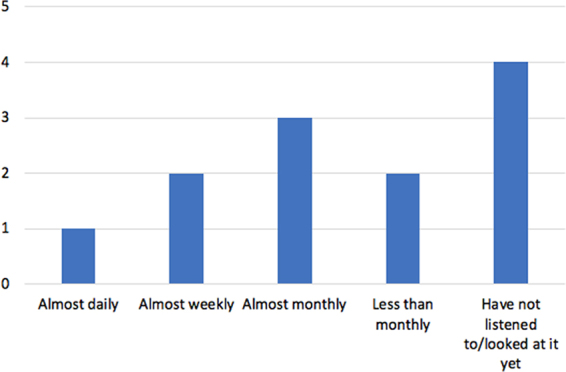
Graph showing how often families either listen to or look at their child's heartbeat recording.

The families varied in how often they looked at or listened to their child's heartbeat recording (Fig. 4). Twenty-five percent (*N* = 3) viewed or listened to the heartbeat recording monthly, 33% (*N* = 4) not at all, 17% (*N* = 2) almost weekly, 17% less than monthly, and 8% (*N* = 1) daily.

In addition to keeping the song and artwork created by the music therapists and child life specialists, four families either had used or planned to use the phonogram of their child's heartbeat as a model for permanent body artwork.

All of the families indicated that they would recommend the MTHS program to other families facing end-of-life decision making. In response to the free-text option at the end of the survey, some families elaborated on how the heartbeat recordings were meaningful to them. Most focused on preservation of memories with their child.

[My daughter] was my center and we would lay and hold her close to me. The heartbeat recording allows me to live in that memory whenever I want to. Thank you for that endless gift.

I love having my son's heartbeat in a song we always sang together.

Do anything we can to preserve the short time we had with him. Thank you all.

I will still be able to hear my son's heartbeat even though he has passed.

It keeps her here with me.

I love having his heartbeat recording to remind me of the tiny heart that changed my life forever. Even though I don't have my sweet [son], I will forever be able to listen to his heart beat.

Hearing my son's heartbeat helps in my healing.

## Discussion

The MTHS program was a simple and cost-effective way to perform legacy making. Implementation was also straightforward and could be completed in a single session. Families who wished to take an active role in the activity were able to contribute with messages from family members or pointing out a meaningful song that could later be added to the recording. For the canvas printed version of the phonocardiogram, they also were able to express themselves by helping to choose color patterns and themes. Creative additions of birth dates, handprints, and family photos helped round out the decoration and bring individual meaning for each bereaved parent. Follow-up sessions were offered for families who wanted to create original music or who wanted to design their own personal artwork to embellish the heartbeat recording.

Consideration for implementation of an MTHS program should focus on several key elements, first being ease of operation. Although this study had access to a music therapist, any member of the medical staff could record the heart sound of the child using the digital stethoscope. One of the benefits of the Eko CORE system is its familiarity to medical practitioners. The system is added to the practitioner's own native stethoscope, and through simple Bluetooth compatibility the .wav and .pdf files are immediately available online when the recording is saved. Heartbeats are able to be printed out minutes after the recording is completed. A second consideration should be given to cost, especially for large health systems who see many patients per year. As a reference point, our hospital pediatric intensive care unit admits roughly 1700 patients per year. Pediatric mortality is considerably lower than in adults; thus, this study was able to function with only one Eko CORE unit priced at less than 200 dollars. Finally, the music therapy team used prepackaged editing software (GarageBand, Apple Inc., Cupertino, CA) to edit the recordings as well as add in family song requests to the background, and the art therapy department supplied canvas and color to create phonocardiogram wall art.

The program was well received by all bereaved parents who said they would recommend it to other families, which is consistent with previous literature. This study also provided a unique opportunity to allow parents to express the impact of the program in their own words. It was similar to the parental response seen in a study of 3D-printed hand molds by Weaver et al., in which the parent involved noted, “This lets us touch a piece of that memory of her in a very real way… Knowing part of her memory is there will feel like a very comforting feeling for our family.”^23^ As the end of life approaches, families often look at the heart monitors to hear or see their child's heart beat for the last few times. Preservation of these intimate sounds and sights provided a unique way for bereaved families to maintain an ongoing connection to their child.

This study also addresses several gaps in the literature. Many studies have looked at legacy making specifically in pediatric oncology patients.^3,19,22,24^ However, this study involves patients who died from both oncologic and nononcologic conditions allowing for more generalizability of the positive effect of legacy making to all patients undergoing end-of-life care.

The current literature also lacks evidence about how legacy making is presented to parents.^3^ In this study, most bereaved parents heard about the MTHS program from a physician or therapist. However, given the time spent and bond formed between patients and their bedside nurses, this study suggests education of nurses about legacy making and the MTHS program may allow more families to benefit from it.

Finally, several studies have looked at the immediate effect of legacy making.^19,20^ In this study, the average amount of time elapsed from the child's death to survey completion was seven months and suggests that legacy making can be part of a continuum of the grief process. Given the evolution of grief over time and the support provided to families by the MTHS program as a resource they can refer back to whenever they need it, there is the potential that the institution of an MTHS program could provide lasting impact at multiple stages of the bereavement process.

There are limitations to this study. First, the small sample size may affect the generalizability to the population as a whole. In addition, our survey consisted of five questions and did not directly ask its effect on bereavement. More direct questions may be needed to fully understand how this intervention affects the bereavement process. The survey nature of the study also relies on parents' recollection of events, which introduces the possibility for error in the form of recall bias. In addition, exclusion of families whose child passed away recently (less than four months ago) may have affected the results. This amount of time was chosen arbitrarily in fear that families who had recently lost a loved one would experience too much pain by being reminded of that loss by early contact from the hospital in the form of a survey. However, grief and the time needed to work through the stages of loss may be different for each individual. For some respondents, four months may not have provided enough time, whereas others may wonder why they were not eligible to be part of the study sooner. Finally, most of the respondents in the survey were the mothers of the children. This may not be generalizable to male parents, and more work is required to understand how the MTHS program may have been of benefit to them.

Further research is needed to better understand how this project affects the bereavement process. Investigating how families use the MTHS program years after the passing of their child and their perception about its role in their bereavement process could answer questions regarding its long-term effect in keeping a deceased child's memory alive. In addition, more studies that investigate why certain families refuse legacy-making activities are needed. As the loss of a child affects the entire family, examining how the MTHS program and the projects created affect siblings may also be beneficial.

## Conclusion

With this evidence from 12 parents, pediatric health systems should consider implementing this low-cost simple legacy-making intervention into the care of seriously ill children. Although the end of a child's life is devastating, offering legacy making has the potential to provide bereaved parents a meaningful and lasting way by which to remember their child.

## Authors Contributions

Ms. Andrews drafted the article. All authors contributed to revisions and overall article content. All authors approved the final article.

## Abbreviation Used

MTHS: Music Therapy Heart Sounds

## References

[1] Middleton W, Raphael B, Burnett P, Martinek N: A longitudinal study comparing bereavement phenomena in recently bereaved spouses, adult children and parents. Aust N Z J Psychiatry 1998;32:235–241 9588303 10.3109/00048679809062734

[2] Lichtenthal WG, Corner GW, Sweeney CR, et al.: Mental health services for parents who lost a child to cancer: If we build them, will they come? J Clin Oncol 2015;33:2246–2253 26033819 10.1200/JCO.2014.59.0406 PMC4486343

[3] Schaefer MR, Spencer SK, Barnett M, et al.: Legacy artwork in pediatric oncology: The impact on bereaved caregivers' psychological functioning and grief. J Palliat Med 2019;22:1124–1128 30892150 10.1089/jpm.2018.0329

[4] Kreicbergs UC, Lannen P, Onelov E, Wolfe J: Parental grief after losing a child to cancer: Impact of professional and social support on long-term outcomes. J Clin Oncol 2007;25:3307–3312 17664479 10.1200/JCO.2006.10.0743

[5] Arnold J, Gemma PB, Cushman LF: Exploring parental grief: Combining quantitative and qualitative measures. Arch Psychiatr Nurs 2005;19:245–255 16308124 10.1016/j.apnu.2005.07.008

[6] Morris S, Fletcher K, Goldstein R: The grief of parents after the death of a young child. J Clin Psychol Med Settings 2019;26:321–338 30488260 10.1007/s10880-018-9590-7

[7] Zetumer S, Young I, Shear MK, et al.: The impact of losing a child on the clinical presentation of complicated grief. J Affect Disord 2015;170:15–21 25217759 10.1016/j.jad.2014.08.021 PMC4253869

[8] Xiu D, Maercker A, Woynar S, et al.: Features of prolonged grief symptoms in Chinese and Swiss bereaved parents. J Nerv Ment Dis 2016;204:693–701 27253073 10.1097/NMD.0000000000000539

[9] Pohlkamp L, Kreicbergs U, Prigerson HG, Sveen J: Psychometric properties of the Prolonged Grief Disorder-13 (PG-13) in bereaved Swedish parents. Psychiatry Res 2018;267:560–565 29982112 10.1016/j.psychres.2018.06.004

[10] Meert KL, Shear K, Newth CJ, et al.: Follow-up study of complicated grief among parents eighteen months after a child's death in the pediatric intensive care unit. J Palliat Med 2011;14:207–214 21281122 10.1089/jpm.2010.0291 PMC3037801

[11] Killikelly C, Maercker A: Prolonged grief disorder for ICD-11: The primacy of clinical utility and international applicability. Eur J Psychotraumatol 2018;8:1536288 29887976 10.1080/20008198.2018.1476441 PMC5990943

[12] American Psychiatric Association: Diagnostic and Statistical Manual of Mental Disorders: DSM-5. Arlington, VA: American Psychiatric Publishing, 2013

[13] Clements-Cortés A: Development and efficacy of music therapy techniques within palliative care. Complement Ther Clin Pract 2016;23:125–129 25986297 10.1016/j.ctcp.2015.04.004

[14] Bradt J, Dileo C, Magill L, Teague A: Music interventions for improving psychological and physical outcomes in cancer patients. Cochrane Database Syst Rev 2016:CD006911 27524661 10.1002/14651858.CD006911.pub3

[15] O'Callaghan C: Bringing music to life: A study of music therapy and palliative care experiences in a cancer hospital. J Palliat Care 2001;17:155–160 11816755

[16] Foster TL, Dietrich MS, Friedman DL, et al.: National survey of children's hospitals on legacy-making activities. J Palliat Med 2012;15:573–578 22577785 10.1089/jpm.2011.0447 PMC3353751

[17] Butler A, Hall H, Willetts G, Copnell B: Parents' experiences of healthcare provider actions when their child dies: An integrative review of the literature. J Spec Pediatr Nurs 2015;20:5–20 25443391 10.1111/jspn.12097

[18] Riegel M, Randall S, Buckley T: Memory making in end-of-life care in the adult intensive care unit: A scoping review of the research literature. Aust Crit Care 2019;32:442–447 30661868 10.1016/j.aucc.2018.12.002

[19] Chochinov HM, Hack T, Hassard T, et al.: Dignity therapy: A novel psychotherapeutic intervention for patients near the end of life. J Clin Oncol 2005;23:5520–5525 16110012 10.1200/JCO.2005.08.391

[20] Akard TF, Dietrich MS, Friedman DL, et al.: Digital storytelling: An innovative legacy-making intervention for children with cancer. Pediatr Blood Cancer 2015;62:658–665 25586983 10.1002/pbc.25337 PMC4339662

[21] Robb SL, Burns DS, Stegenga KA, et al.: Randomized clinical trial of therapeutic music video intervention for resilience outcomes in adolescents/young adults undergoing hematopoietic stem cell transplant: A report from the Children's Oncology Group. Cancer 2014;120:909–917 24469862 10.1002/cncr.28355 PMC3947727

[22] Akard TF, Gilmer MJ, Friedman DL, et al.: From qualitative work to intervention development in pediatric oncology palliative care research. J Pediatr Oncol Nurs 2013;30:153–160 23632900 10.1177/1043454213487434 PMC3808110

[23] Weaver MS, Linke G, Robinson J, Wratchford D: All that this hand may hold: Phenomenological exploration into the meaning of pediatric legacy prints. J Pain Symptom Manage 2020;59:761–765 31200014 10.1016/j.jpainsymman.2019.05.016

[24] Beiermann M, Kalowes P, Dyo M, Mondor A: Family members' and intensive care unit nurses' response to the ECG memento© during the bereavement period. Dimens Crit Care Nurs 2017;36:317–326 28976481 10.1097/DCC.0000000000000269

